# Prediction of pathological response grading for esophageal squamous carcinoma after neoadjuvant chemoradiotherapy based on MRI imaging using PDX

**DOI:** 10.3389/fonc.2023.1160815

**Published:** 2023-06-12

**Authors:** Jingzhen Shi, Jianbin Li, Zhenxiang Li, Yankang Li, Liang Xu, Yingjie Zhang

**Affiliations:** ^1^ Department of Oncology, The Second Hospital of Tianjin Medical University, Tianjin, China; ^2^ School of Medicine, Shandong University, Jinan, China; ^3^ Department of Radiation Oncology, Shandong Cancer Hospital and Institute, Shandong First Medical University and Shandong Academy of Medical Sciences, Jinan, China; ^4^ Department of Medical Imaging, Shandong Cancer Hospital and Institute, Shandong First Medical University and Shandong Academy of Medical Sciences, Jinan, China

**Keywords:** patient-derived xenograft, esophageal squamous cell carcinoma, magnetic resonance-diffusion weighted imaging, pathological response grading, neoadjuvant chemoradiotherapy

## Abstract

**Introduction:**

To confirm the efficacy of magnetic resonance-diffusion weighted imaging (MR-DWI) in esophageal squamous cell carcinoma (ESCC) early pathological response prediction and assessment to neoadjuvant chemoradiotherapy (nCRT) using patient-derived xenografts (PDXs)

**Methods:**

PDX-bearing mice were randomly divided into two groups: the experimental group receiving cisplatin combined with radiotherapy, whereas the control group receiving normal saline. MRI scans were performed in treatment groups in the before, middle, and end of treatment. The correlations between tumor volumes, ADC values and tumor pathological response at different time nodes were explored. Then, expression of proliferation marker and apoptotic marker were detected using immunohistochemistry, and apoptosis rate was detected by TUNEL assay to further verify the results observed in the PDX models.

**Results:**

The ADC values of the experimental group were significantly higher than the control group in the both middle and end stage of treatment (all *P*< 0.001), however, significant difference was only observed in tumor volume at the end stage of treatment (P< 0.001). Furthermore, the △ADC_mid-pre_ in our study may able to identify tumors with or without pCR to nCRT at an early stage, due to these changes were prior to the changes of tumor volume after treatment. Finally, TUNEL results also showed that the apoptosis rate of the experiment groups increased the most in the middle stage of treatment, especially the groups with pCR, but the highest apoptosis rate occurred in the end of the treatment. Further, the two PDX models with pCR exhibited the highest levels of apoptotic marker (Bax), and lowest levels of proliferation marker (PCNA and Ki-67) in the both middle and end stage of the treatment.

**Conclusions:**

ADC values could be used to determine the tumor’s response to nCRT, especially in the middle stages of treatment and before the tumor tissue morphology changes, and further, the ADC values were consistent with the potential biomarkers reflecting histopathological changes. Therefore, we suggest that radiation oncologists could refer to the ADC values in the middle stages of treatment when predicting the tumor histopathological response to n CRT in patients with ESCC.

## Background

According to the 2015 China Cancer Report, esophageal cancer (EC) ranks sixth in cancer incidence and fourth in cancer mortality, with 246,000 new esophageal cancer cases and 188,000 deaths in China. Esophageal squamous cell carcinoma (ESCC), accounting for more than 95% of all cases of EC, was deemed to the predominant histological type in China (1, 2). Most patients with EC are initially diagnosed at locally advanced stages. At this time, the effect of surgery alone is not good for these patients: the 5-year overall survival (OS) rate between 15%-24% (3–5). Due to the little benefit, the combination of radiotherapy, chemotherapy and surgery to improve the efficacy of operable esophageal cancer has attracted worldwide attention, and neoadjuvant chemoradiotherapy(nCRT) combined with surgery is still a hotspot in current study.

Unfortunately, not all patients with ESCC benefit from nCRT, only the patients with ESCC would benefit from nCRT when achieving pathological complete response(pCR), significantly (6, 7). The prediction of the pathological response in the early stage of treatment could contribute to make treatment decisions and improve the treatment outcome for the operable ESCC. Therefore, it is urgent for us to identify the ESCC patients who will benefit from nCRT so that the best treatment can be given to each individual patient. However, there is still no concensus precise model for predicting the pathologic response grading for ESCC.

Magnetic resonance-diffusion weighted imaging (MR-DWI), as a new technique in molecular functional imaging, could detect microenvironmental changes in tumors at an early stage. These changes were preceding the morphological changes of tumors, and shown advantages in assessing the near-term efficacy (8). Various tissues have unique diffusion characteristics, as measured by the apparent diffusion coefficient (ADC), which can be calculated from the diffusion-weighted imaging (DWI) measurements (9). ADC values depend on the impediment to free diffusion of water molecules in a single voxel due to restrictive barriers such as fibers, membranes, and macromolecules in tissue compartments. Usually, due to the rapid growth of malignant tumors and their dense cells, the free movement of water molecules inside and outside the cell was limited and the ADC value was low. On the other hand, effective antitumor therapy could affect the permeability and integrity of cell membrane, resulting in lower tumor density and higher ADC value (10, 11). Although previous studies have demonstrated that DWI could detect metastatic lymph nodes and tumor in EC (12, 13), no reports have been published on the efficacy of DWI in calculating ADC to predict the nCRT response using Patient-derived xenograft (PDX) models of ESCC.

Recently, PDX models, which are established by transplanting tumors from cancer patients directly into immunocompromised mice, have been used to evaluate the efficacy of targeted treatments for different types of tumors, such as those of breast cancer and non-small cell lung cancer (NSCLC) (14, 15). The models may more accurately represent a tumor in a real patient because they can retain the genetic information, immunohistological markers and chemosensitivity of the primary tumor through a few passages. Therefore, PDX models have an advantage over cell line-based xenograft models (16–19). Indeed, these models are becoming the preferred preclinical tool used to study specific anticancer therapies for cancer patients as well as biological and genetic alterations (20).

In this study, we successfully established PDX models of ESCC from tumor tissues to confirm the use of the ADC values in ESCC early pathological response prediction and assessment to nCRT.

## Materials and methods

### Patients and tissue samples

This study was approved by the research ethics board of our institute, and written informed consent for the use of the tissue samples and the publication of any potentially identifiable images or data was obtained from every patient before enrollment in the study. Between December 2019 and September 2020, 18 patients were enrolled in this study. All patients in this study had pathologically proven ESCC and available tissue, and none had been scheduled to undergo radiotherapy or chemotherapy before surgery. Tumor tissue was obtained intraoperatively from the edge of the whole tumor mass and transported to the animal laboratory in transport medium (fetal bovine serum (FBS)-free RPMI-1640 medium supplemented with penicillin and streptomycin) under sterile conditions.

### Establishment of the PDX models

The tumor samples were rinsed in a Petri dish containing FBS supplemented with penicillin and streptomycin and carefully cut into small fragments (approximately 30 mm3). Then, the fragments were subcutaneously implanted into five male NOD-SCID mice (5 weeks old) obtained from Beijing Vital River Laboratory Animal Technology Co., Ltd. Additional tumor tissue was cryopreserved (90% FBS and 10% DMSO) and snap frozen in liquid nitrogen for future use. All procedures were performed in a certified biosafety hood and carried out in complete accordance with protocols approved by the Committee for the Care and Use of Laboratory Animals of our institute. Xenografts were assessed by palpation and Vernier calipers at least twice weekly for up to six months. The successfully established PDX model was named passage 1 (P1). Mice were kept until the tumor volume reached 700-800 mm3 if the implanted tumors grew. At that point, mice were decapitated and the tumor samples were re-implanted into other mice following the same protocol as previously described. Subsequent passages were sequentially named P2, P3, and P4. The remaining tissues were stored at -80°C for subsequent detection and future usage.

### Treatment

When the tumors reached 100 mm3, 44 mice bearing P4 xenografts from 4 patients were randomly divided into two groups as follows: the experimental group receiving cisplatin combined with radiotherapy (8 Gy×4, n=22), whereas the control group receiving normal saline (n=22). Mice in the treatment groups were anesthetized and subjected to local irradiation (8 Gy) to the tumors once a week for a total of 4 times. Cisplatin (2 mg/kg) was administered intraperitoneally once weekly for 4 weeks. MRI scans were performed sequentially in treatment groups on day 0 (day 1 prior to chemoradiotherapy), 15 days (day 15 after chemoradiotherapy), and 29 days (day 29 after chemoradiotherapy) in return. After MRI irradiation, Tumor- bearing xenografts were immediately executed (cervical vertebral). Tumor volumes and body weights were measured 2 times weekly. The tumor volume measurements performed on T2-weighted MR images using MIM software (MIM 6.8.5; MIM Software Inc., Cleveland, OH, USA). The tumor volume before chemo-radiotherapy was named V_pre_, tumor volume during treatment was named V_mid_, and that after treatment was named V_end_.

### MRI image acquisition

**Figure 1:**
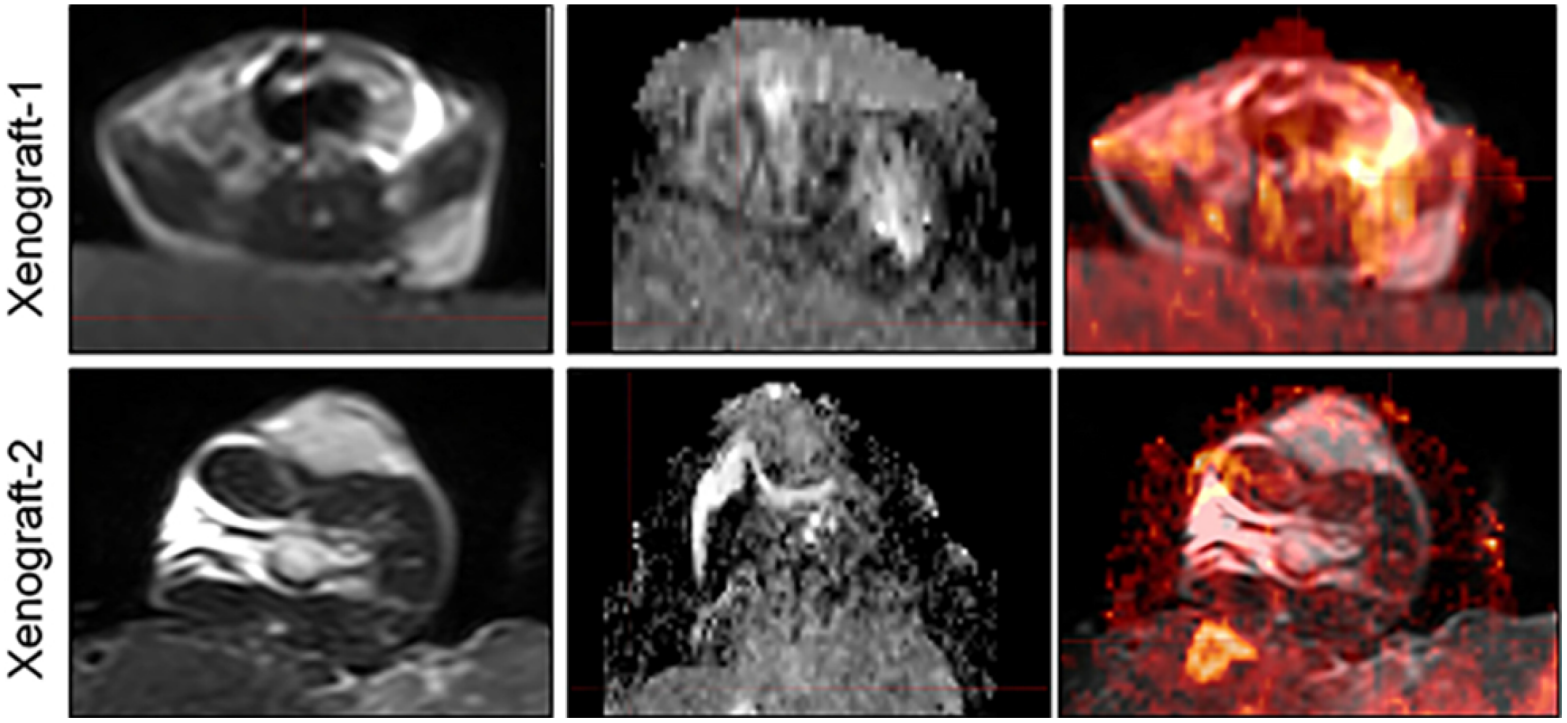
Representative images of MRI and ADC, as well as the deformed images using DIR.

Prior to MR imaging, the PDX mice were anaesthetized with 3% pentobarbital injections (0.3 ml/mouse). Then the mice were wrapped with fresh pork and placed in loop coils. Scans were conducted on a clinical 3.0T MRI scanner (INGENIA, PHILIPS Roval, Amsterdam, Netherlands). T2WI, and DWI sequences were included in our study. DWI images were acquired using an HD dispersion sequence at b- values (dispersion- sensitive gradient) of 0 and 600 s/mm2 (21–23), and the ADC images would be generated on a postprocessing workstation subsequently. In our study, we also provided the deformed images of two xenografts to demonstrate the accuracy of DIR in MIM software (**Figure 1**).

### Measurement of ADC

The T2W images and ADC images were transferred to MIM software (MIM 6.8.5; MIM Software Inc., Cleveland, OH, USA). The tumors were first manually contoured on the new deformed image derived from T2W images and ADC images by MIM software, following the contours would be transfer to the ADC images auto-manually, and then contours were specifically selected by clicking the green Checkmark Button at the right edge of the viewport. Later, the average ADC value within a specific outlined tumor would be derived directly by the system’s statistics viewer. The ADC value before chemo-radiotherapy was named ADC_pre_, ADC value during treatment was named ADC_mid_, and that after treatment was named ADC_end_.

### H&E staining and immunohistochemistry

For histopathological assessment, primary tumors and xenografts were embedded in paraffin blocks and then stained with hematoxylin and eosin (H&E). All tissue sections were stained with an H&E staining kit (C0105, Beyotime, China) after the 5-µm sections were deparaffinized with dimethylbenzene according to a standard method and were evaluated by two independent pathologists.

For IHC, tissue sections were deparaffinized and hydrated. After antigen retrieval with sodium citrate antigen retrieval solution (pH=6.0) and blocking with 3% BSA, sections were hybridized with a primary antibody (specific for c-PARP, BAX, PCNA) overnight at 4°C. Then, an HRP-conjugated secondary antibody (recognizing the appropriate primary antibody species) was added and incubated at room temperature for 50 min. Tissue sections were developed with freshly prepared DAB chromogenic reagent and counterstained with hematoxylin staining solution for 3 min. Finally, sections were dehydrated successively in a graded series of 75%, 85%, and 100% ethanol and mounted with resin mounting medium. Nuclei stained with hematoxylin appear blue, and positive cells developed with DAB reagent appear brownish yellow. The results were obtained based on the average of any four fields in 200 times. All sections were observed by microscopy and analyzed using the Image-Pro Plus 6.0 software program (Media Cybernetics, Rockville, MD, USA).

### TUNEL assay

Tissue sections were deparaffinized and rehydrated. After antigen retrieval with proteinase K working solution and permeabilization with permeabilization working solution, a mixture of TdT and dUTP at a ratio of 1:9 was added to the slides and incubated at 37°C for 2 h for the TUNEL reaction. Then, endogenous peroxidase activity was blocked, and the tissues were covered with reagent 3 (converter-POD). Freshly prepared DAB chromogenic reagent was added to the tissue sections, which were then counterstained with hematoxylin staining solution for 3 min. Finally, the sections were dehydrated successively in a gradient of 70%, 80%, 95%, and 100% ethanol followed by xylene and were mounted with resin mounting medium. Nuclei stained with hematoxylin appear blue, and positive cells developed with DAB reagent appear brownish yellow. All sections were observed by microscopy and analyzed using the Image-Pro Plus 6.0 software program (Media Cybernetics, Rockville, MD, USA).

### Statistical analysis

Data from skewed distributions were presented as the M (median) and IQR (inter-quartile range). Statistical analyses were performed using Wilcoxon test to compare the differences between two groups in SPSS 22.0 software (IBM, USA). The degree of association between the ADC value, volume of tumor and pCR were calculated by the Spearman test. *P* < 0.05 was considered statistically significant.

## Results

To investigate the correlation between the ADC values, volumes of tumor and pathological response in PDX models, tumor regression of PDX models were divided into two groups according to seventh edition of the American Joint Committee On Cancer (AJCC): group A, including two PDX models with pCR after treatment; group B, including other two PDX models with pathologic partial reponse after treatment.

### Patient characteristics

Clinical characteristics of studied patients were shown in **Table 1**. A total of 18 samples of ESCC (4 in the upper segment, 9 in the middle segment and 5 in the distal segment) were obtained by surgical resection. Ultimately, 4 PDX models were successfully established in NOD/SCID mice. **Table 1** lists the baseline characteristics of the subjects. The donor patients included 14 men (78%) and 4 women (22%) with a median age of 61.5 years (range, 46-74). Nine patients had lymph node (LN) metastasis, and no patient had been treated before surgery. According to the AJCC 8th staging system, nine (50%) patients were diagnosed with stage II disease, and nine patients (50%) were diagnosed with stage III/IV disease. Among these patients, 4 samples (22%) were well differentiated, 10 samples (56%) were moderately differentiated, and 4 samples (22%) were poorly differentiated.

**Table 1:** Clinical characteristics of studied patients.

NO.	Gender	Age (years)	Tumor differentiation	TNM staging	Tumor location	Engrafted mode
EG1	Male	69	Moderate	T3N0M0 IIB	Distal	No
EG2	Male	67	Well	T3N1M0 IIIB	Middle	No
EG3	Male	69	Well	T4N2M0 IVA	Distal	Yes
EG4	Male	74	Moderate	T2N0M0 IIA	Distal	No
EG5	Female	58	Moderate	T3N1M0 IIIB	Middle	No
EG6	Male	58	Poor	T3N2M0 IIIB	Distal	Yes
EG7	Male	71	Poor	T3N0M0 IIB	Middle	No
EG8	Male	46	Moderate	T3N1M0 IIIB	Middle	Yes
EG9	Male	67	Moderate	T3N0M0 IIB	Middle	No
EG10	Female	62	Moderate	T3N0M0 IIB	Middle	No
EG11	Male	56	Well	T2N2M0 IIIB	Middle	No
EG12	Male	56	Poor	T3N0M0 IIA	Middle	No
EG13	Female	58	Well	T3N0M0 IIA	Upper	No
EG14	Male	58	Moderate	T3N1M0 IIIA	Upper	No
EG15	Male	61	Moderate	T3N0M0 IIB	Middle	No
EG16	Female	63	Moderate	T3N0M0 IIA	Distal	No
EG17	Male	59	Poor	T3N1M0 IIIA	Upper	Yes
EG18	Male	65	Moderate	T2N2M0 IIIB	Upper	No

### Establishment of PDX models

Four transplanted xenografts were successfully passaged to the fourth generation (range, 6.1 months-10 months), and the overall transplantation rate for PDXs was 22% (4/18) in our study. The transplantation rate was increased along with serial passaging: 28% (5/18), 80% (4/5), 100% (4/4), and 100% (4/4) for P1, P2, P3, and P4 PDXs, respectively. Additionally, the median latency period for the third passage was shorter than the first and second passage PDXs: 78.56 ± 19.21 days, 55.67 ± 16.52 days and 29.96 ± 15.47 days for P1, P2, and P3, respectively. After the fourth generation, the PDX models exhibited robust growth and stability without further changes in model formation; thus, they could be used in future studies.

### The histology was consistent between the original patient tumors and the xenografts

**Figure 2:**
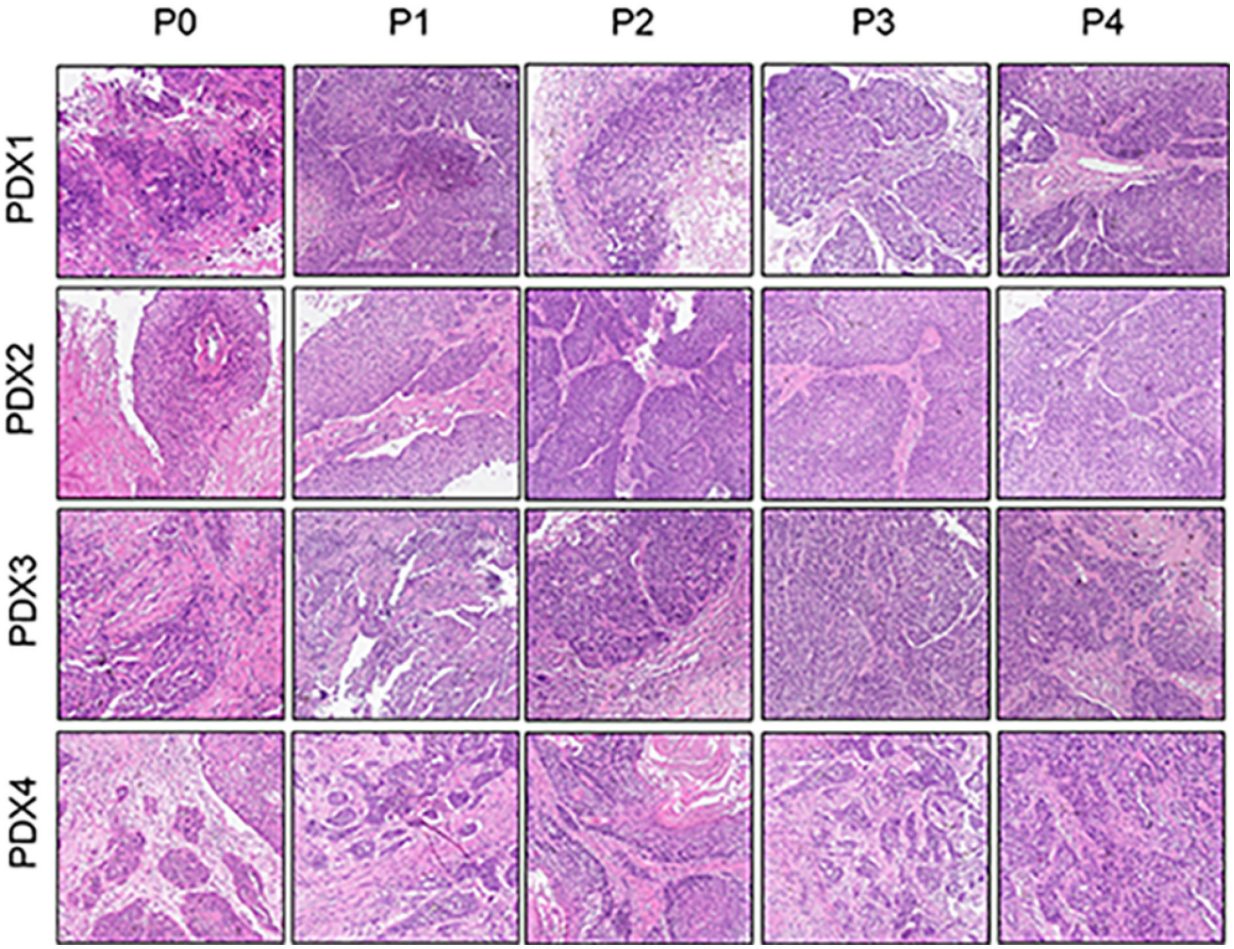
Comparative histological section(HE) of the esophageal patient tissue(P0) with the series passage xenografts (P1,P2,P3,P4) to verify the histologistical characteristics of tumor tissues (x200).

**Figure 3:**
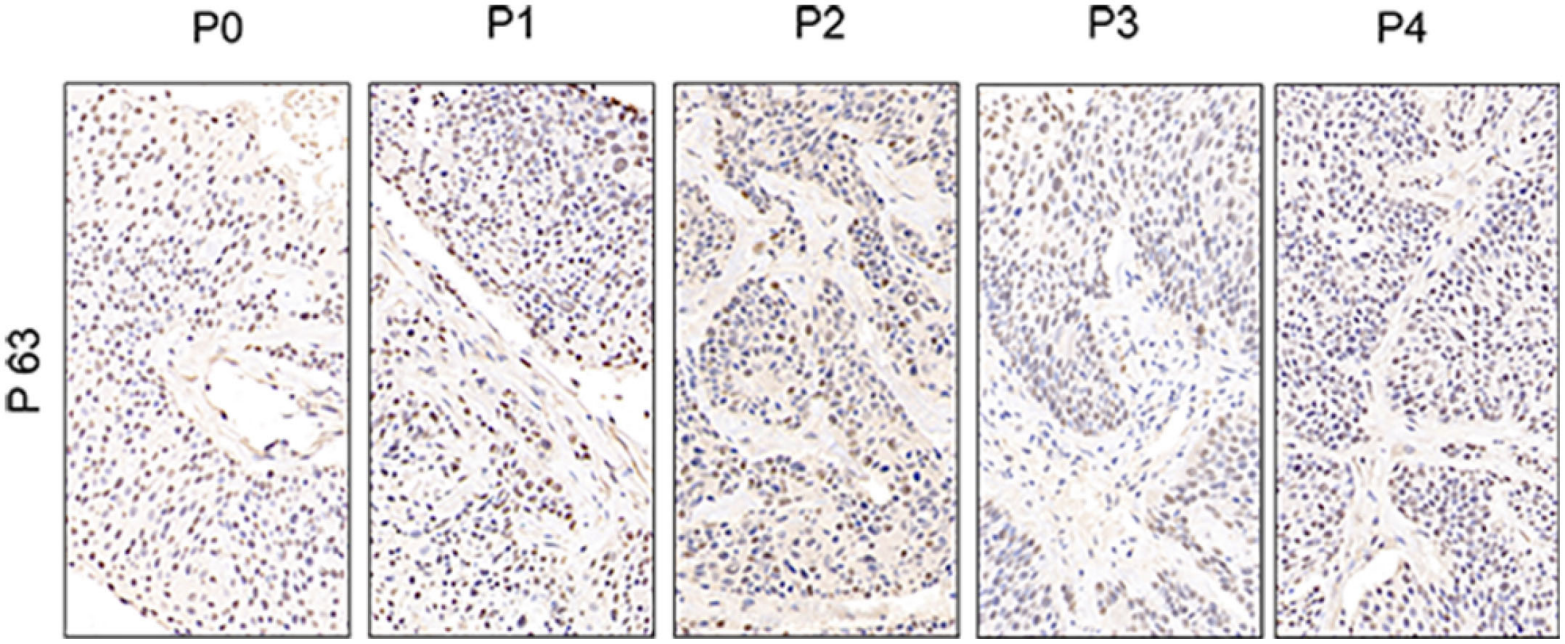
Comparative immunohistochemistry of P63 in the esophageal patient tissue(P0) with the series passage xenografts (P1,P2,P3,P4) to verify the histologistical characteristics of tumor tissues (x400).

To further assess the PDX xenografts, we initially compared the histology of the original patient tumors with that of the corresponding serially passaged xenografts by H&E staining. The pathological characteristics of the passaged xenografts were consistent with those of the original patient tumor tissues (**Figure 2**). In addition, the P63 gene and its encoded protein play important roles in the early physiological and pathological course of disease in the esophageal mucosa and may be helpful in the early diagnosis and prognostic evaluation of ESCC. Our immunohistochemical staining results proved that the expression of P63 was positive in all P4 PDXs, further proving the squamous phenotype of the tumors (**Figure 3**).

### Comparison of the volumes and ADC values of PDX models between the experimental and control groups before, middle and after chemoradiotherapy

Four PDX models were used in our study, and the mice in every model (44 mice in total) were randomly divided into two groups.

The median volume V_pre_ of the control group and the experimental group was 114.79 and 115.85 mm3, respectively, and no statistical significance was observed in these two groups (*P* = 0.884). The median volume V_mid_ of the control group was slightly larger than the experimental group, but there was no statistically significant difference between the two groups (*P* = 0.177). However, we found that the tumor volume V_end_ of the groups (control group was significantly greater than that of the experimental group (*P<* 0.001). Further, we also found the differences in the △V_end-mid_ and △V_end-pre_ values between the control group and the treatment group were significant (all *P<* 0.001). However, no significant difference was observed in △V_mid-pre_ (*P* = 0.158).

The median ADC_pre_ of the control groups and the experimental group were 1.000 and 1.025(x10-6mm2 / s), respectively, and no statistical significance was observed in our study (*P* = 0.741). However, there were significant difference between these two groups in ADC_mid_ and ADC_end_ (all *P<* 0.001). Further, our results also showed that the differences in the △ADC_mid-pre_, △ADC_end-mid_ and △ADC_end-pre_ values between the control group and the treatment group were significant (all *P<* 0.001). The ADC values of the two groups began to show a significant difference in the middle stage of treatment, that is, the ADC values of the experimental group were significantly higher than the control group in the middle stage of treatment (*P<* 0.001). We also found that the ADC values of the experimental group decreased the most from the ADC_pre_ to ADC_mid_, but maximum ADC was reached at the end of treatment in our study.

### Analysis of ADC values or △ADC values in identifying pathological complete response

The results are shown in **Table 2**. In the control group, there was no significant difference in △ADC_mid-pre_ between the group A with pCR and group B without pCR, but significant difference was observed in ADC_pre_, ADC_mid_, ADC_end_, △ADC_end-mid_, and △ADC_end-pre_ between the two groups (all *P*< 0.05). In the experimental group, we found the differences in the ADC_pre_, ADC_mid_, ADCend, △ADC_mid-pre_, △ADC_end-mid_ and △ADC_end-pre_ between the group A with pCR and group B without pCR were all significant (all *P<* 0.05).

**Table 2:** Analysis of ADC values or △ADC values in identifying pathological complete response(×10−6 mm2/s).

ADC (×10^−6^ mm^2^/s)	control group				P	experiment group				P
	group A		group B			group A		group B		
	M	IQR	M	IQR		M	IQR	M	IQR	
ADCpre	1.105(1.078,1.153)		0.920(0.853,0.950)		0.005	1.130(1.060,1.170)		0.910(0.840,0.980)		0.003
ADCmid	0.965(0.888,0996)		0.835(0.803,0.900)		0.012	1.730(1.660,1.780)		1.360(1.311,1.390)		0.013
ADCend	0.910(0.810,0.975)		0.800(0.753,0.830)		0.013	1.910(1.870,1.990)		1.490(1.450,1.520)		0.003
△ADCmid-pre	-0.065(-0.080,-0.030)		-0.070(-0.108,-0.043)		0.722	0.610(0.540,0.640)		0.470(0.380,0.520)		0.003
△ADCend-mid	-0.160(-0.190,-0.145)		-0.055(-0.088,-0.025)		0.003	0.210(0.120,0.280)		0.170(0.070,0180)		0.012
△ADCend-pre	-0.225(-0.268,-0.155)		-0.135(-0.188,-0.085)		0.037	0.820(0.690,0.840)		0.540(0.530,0.666)		0.003

M, median; IQR, inter-quartile range.

### Analysis of tumor volumes or △volumes in identifying pathological complete response

The results are shown in **Table 3**. In the control group, there was a significant difference in the V_end_, △V_end-mid_, and △V_end-pre_ between the group A with pCR and group B without pCR (all *P<* 0.05), However, a significant difference in the V_pre_, V_mid_, and △V_mid-pre_ was not found between these two groups (*P*=0.878,0.237,0.241). In the experimental group, we also found the differences in the V_end_, △V_end-mid_, and △V_end-pre_ between the group A with pCR and group B without pCR were significant (all *P<* 0.05), and the difference in the V_pre_, V_mid_, and △V_mid-pre_ was not found between these two groups (*P*=0.790,0.314,0.790).

**Table 3:** Analysis of tumor volumes or △volumes in identifying pathological complete response(mm^3^).

Volumes (mm^3^)	control group				P	experiment group				P
	group A		group B			group A		group B		
	M	IQR	M	IQR		M	IQR	M	IQR	
V_pre_	115.47 (109.64,121.05)		114.78 (110.71,122.27)		0.878	116.55 (109.11,120.55)		114.48 (110.58,121.51)		0.790
V_mid_	133.74 (130.83,138.79)		139.86 (130.41,144.75)		0.237	132.51 (127.98,137.24)		130.33 (123.45,143.41)		0.314
V_end_	210.68 (198.35,220.68)		231.15 (218.36,235.39)		0.013	91.98 (88.79,98.35)		123.49 (115.45,135.41)		0.003
△Vmid-pre	19.50 (10.72,22.71)		23.09 (18.22,25.22)		0.241	15.34 (10.90,20.99)		12.35 (10.00,22.52)		0.790
△Vend-mid	76.93 (68.13,83.05)		89.31 (80.95,104.97)		0.037	-38.78 (-42.65,-35.97)		-7.00 (-16.52,-5.00)		0.003
△Vend-pre	91.54 (85.72,101.84)		117.24 (101.59,126.58)		0.022	-20.79 (-28.94,-15.930		12.89 (2.58,17.16)		0.003

M, median; IQR, inter-quartile range.

### Correlation analysis between ADC value or tumor volume and pathological response

The ADC_mid_, ADC_end_, △ADC_mid-pre_ and △ADC_end-pre_ showed a significant positive correlation with the pathological response for all PDXs (*R*=0.567, 0.868, 0.854, 0.741 all *P*<0.05). Additionally, the V_end_, △V_end-mid_ and △V_end-pre_ showed a significant negative correlation with the pathological response for all PDXs (*R*=-0.853, -0.853, -0.867 all *P*<0.05).

### Comparison of apoptosis and proliferatio of PDX models by TUNEL assay and immunohistochemistry

**Figure 4:**
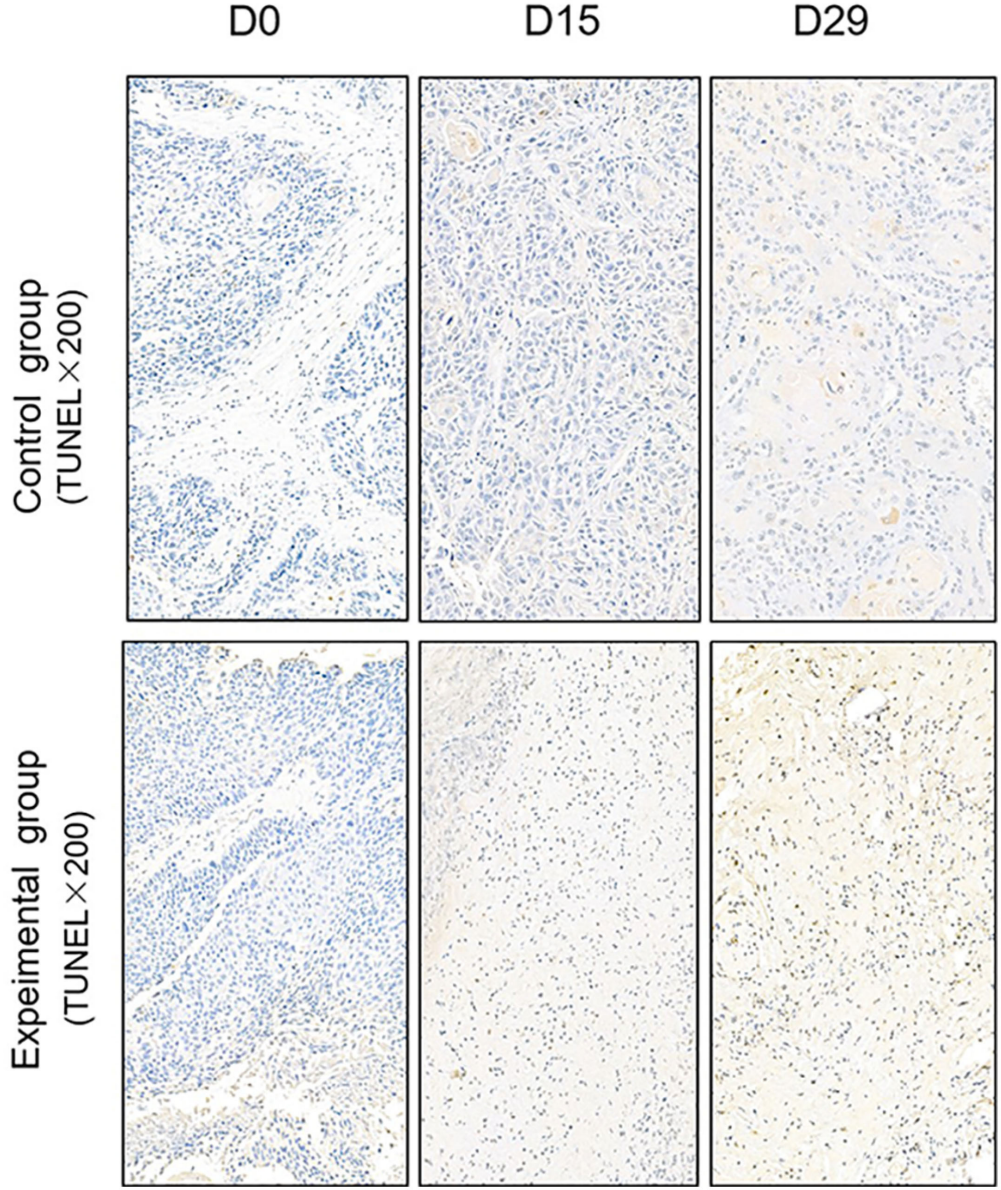
Representative images of Tunnel assay in tumor xenografts of SCID mice(x200; D0: day 1 prior to chemoradiotherapy; D15: day 15 after chemoradiotherapy; D29: day 29 after chemoradiotherapy).

**Figure 5:**
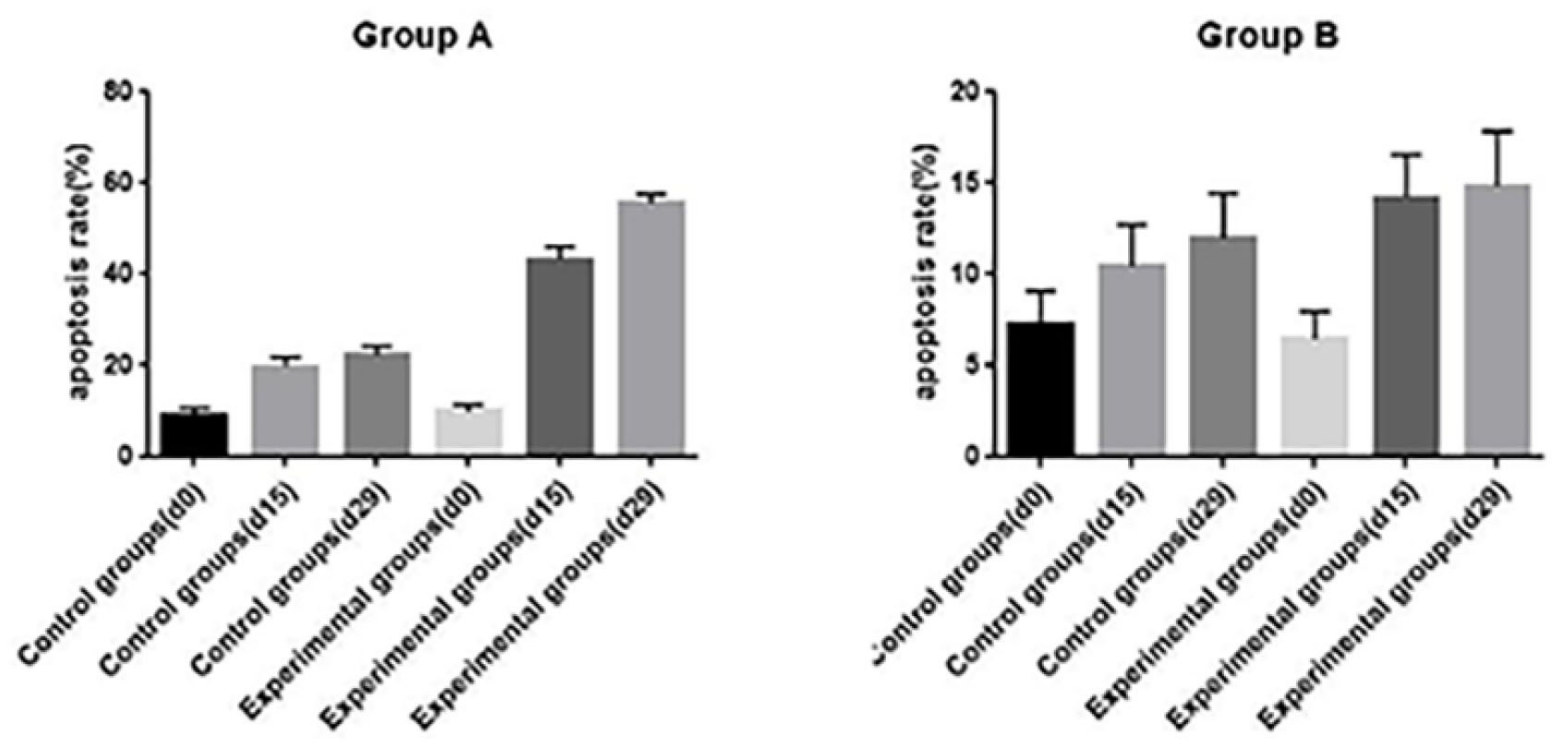
The opoptosis rates of different groups in PDX models(d0: day 1 prior to chemoradiotherapy; d15: day 15 after chemoradiotherapy; d29: day 29 after chemoradiotherapy).

**Figure 6:**
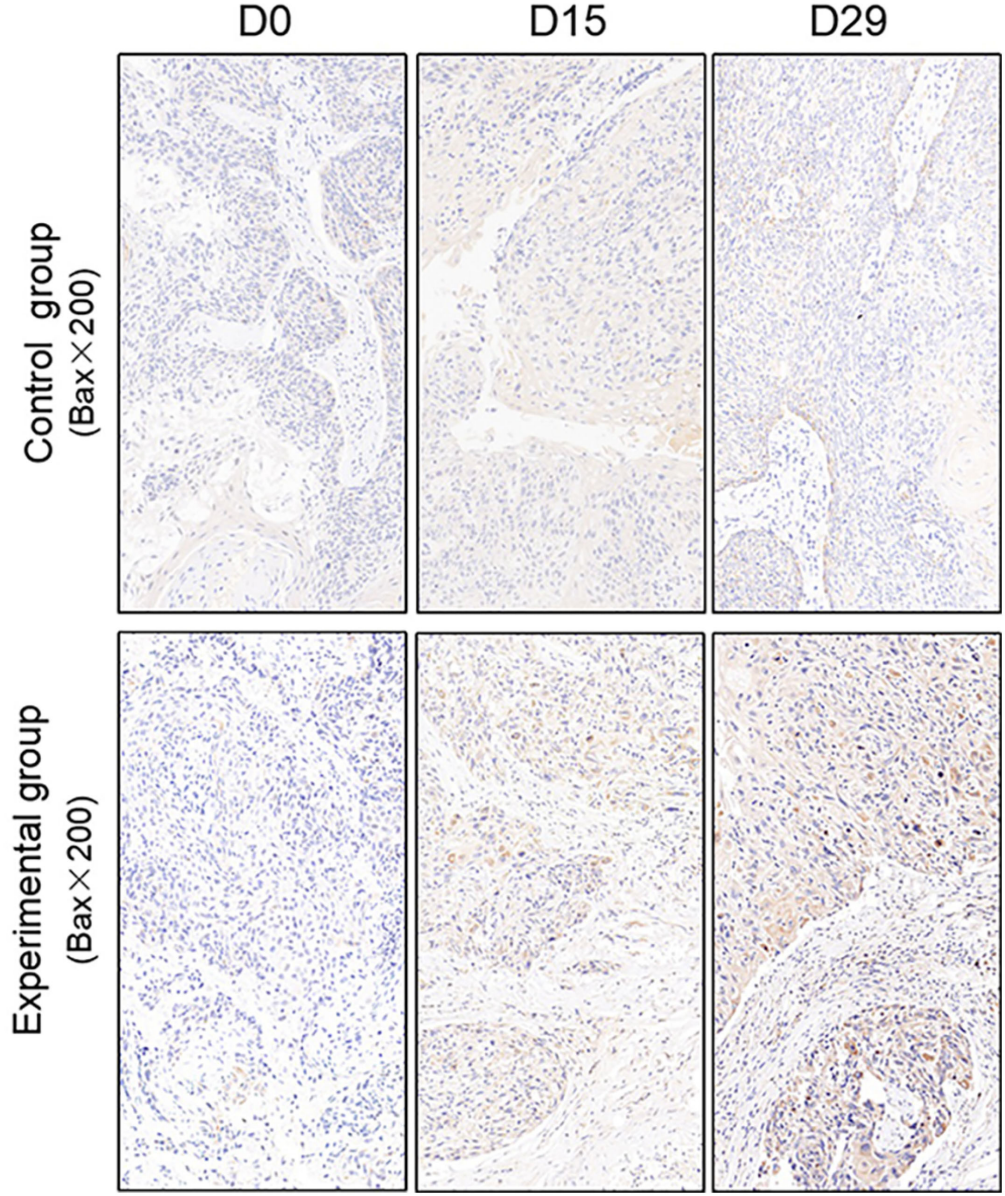
Representative images of Bax in tumor xenografts of SCID mice(x200;Bax, markers for apoptosis; D0: day 1 prior to chemoradiotherapy; D15: day 15 after chemoradiotherapy; D29: day 29 after chemoradiotherapy).

**Figure 7:**
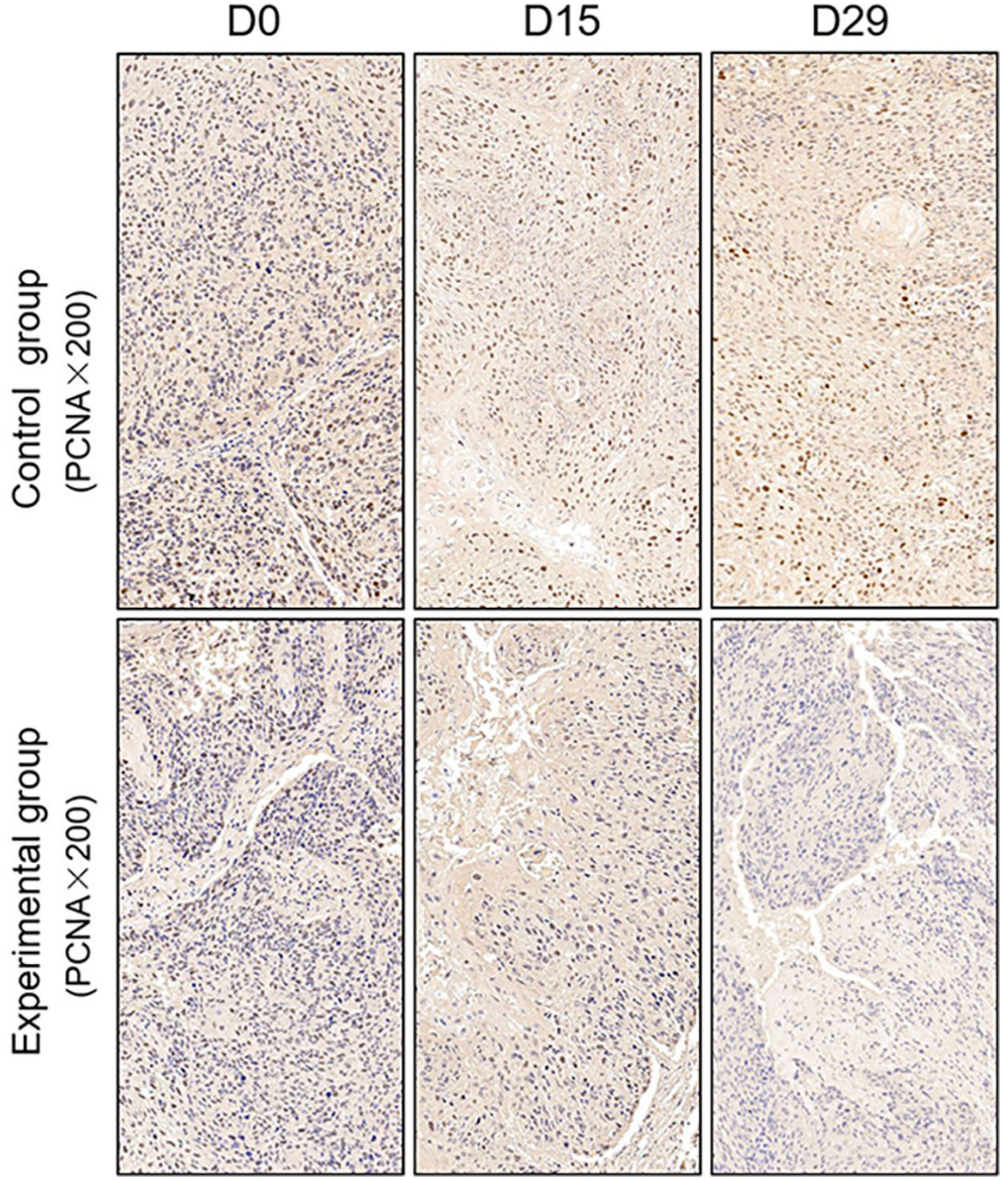
Representative images of PCNA in tumor xenografts of SCID mice(x200;PCNA, markers for Proliferation; D0: day 1 prior to chemoradiotherapy; D15: day 15 after chemoradiotherapy; D29: day 29 after chemoradiotherapy).

**Figure 8:**
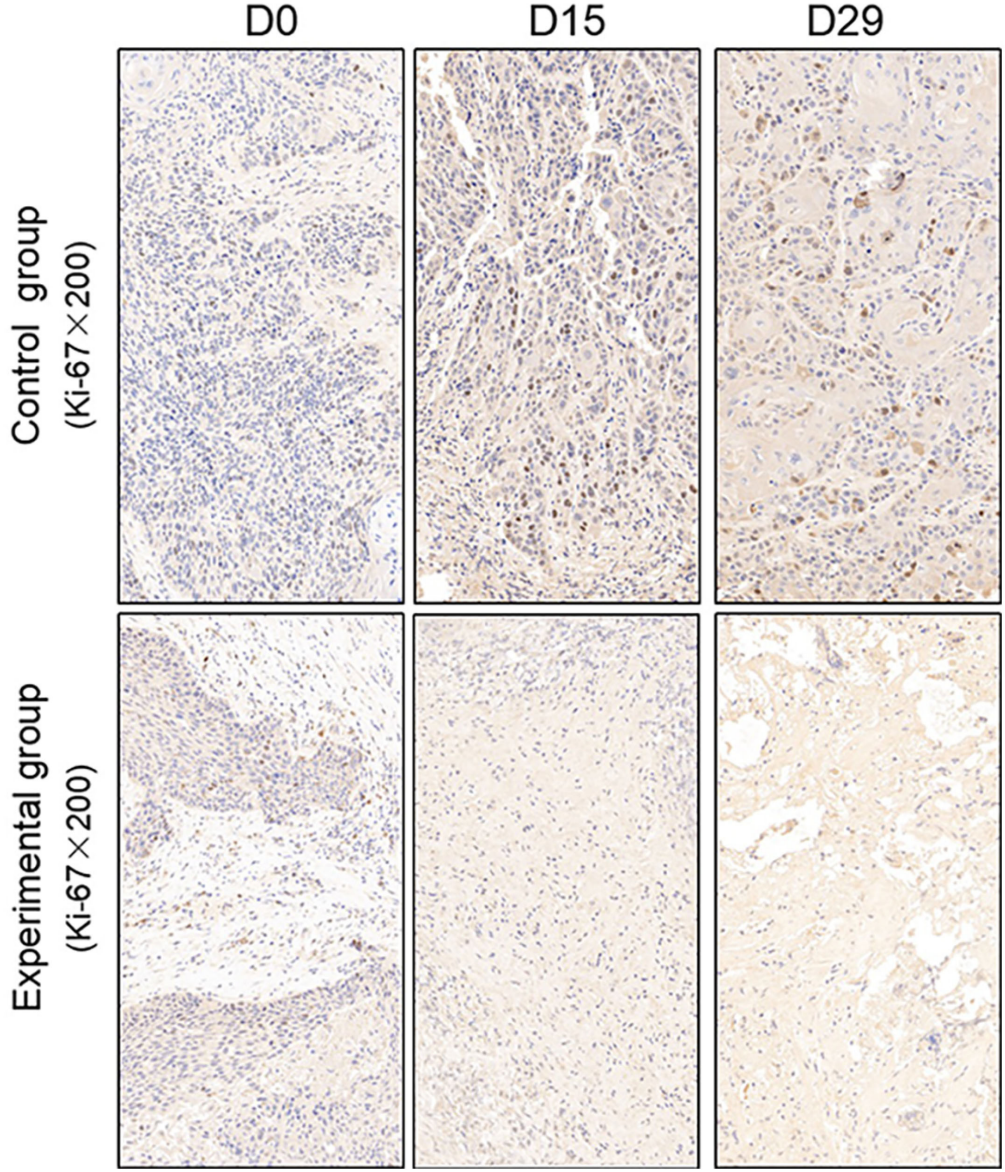
Representative images of Ki-67 in tumor xenografts of SCID mice(x200;Ki-67, markers for proliferation; D0: day 1 prior to chemoradiotherapy; D15: day 15 after chemoradiotherapy; D29: day 29 after chemoradiotherapy).

TUNEL assay and immunohistochemistry (IHC) were performed within first a week after treatment. Firstly, we evaluated apoptosis *via* both TUNEL and apoptotic marker (Bax) immunoreactivity in various groups. The results showed that the apoptosis rate of the experiment groups increased the most in the middle stage of chemoradiotherapy, especially the groups with pCR, but the highest apoptosis rate occurred in the end of the treatment (**Figure 4**). At the same time, we found that the cancer cell apoptosis rate of the experimental groups was higher than that of the control group from the middle stage of treatment (*P* < 0.05). Furthermore, the two PDX models achieving pCR showed higher cancer cell apoptosis compared with the other two PDX models achieving pathological partial response in the both middle and end of treatment (**Figure 5**). The immunohistochemical staining results also showed that the Bax levels increased significantly in the treatment groups compared with the control groups in the middle of the treatment, and the two PDX models with pCR exhibited the highest levels the both middle and end of treatment (**Figure 6**). In addition, cells immunoreactive for the proliferation marker (PCNA and Ki-67) decreased significantly in the treated tumor masses compared with the normal saline-treated tumor masses in the middle of the treatment, and the PDX models with pCR resulted in the lowest PCNA levels and Ki-67 in the both middle and end of treatment (**Figures 7**, **8**).

## Discussion

ESCC is a serious malignancy with a high mortality rate and poor prognosis in China, accounting for more than 95% of cases of EC (1, 2). Preoperative chemoradiotherapy for locally advanced esophageal cancer was recommended to down staging and improve R0 resection, but there still were 30% patients with R0 resection failing in local control, and only patients who achieve pCR have been shown to significantly improve local control rate and tumor-related long-term survival (6, 7). Therefore, in order to achieve precise treatment for patients with EC, it is necessary to predict the pathological response of EC to nCRT, identify the beneficiary population, and reduce the unnecessary treatment.

MR-diffusion weighted imaging (MR-DWI), as a new technology, reflects the diffusion motions of water molecule in the tissue and could be used to evaluate the changes of tumor volume and function. ADC value is a quantitative index of DWI, which is negatively correlated with tissue cell density. When the tissue cell density decreases, the extracellular space increase, the interstitial fluid pressure decreases, the dispersion ability of the water molecule strengthens, the DWI signal decreases, and the ADC value increases (8, 24). Although previous studies have demonstrated that MRI DWI imaging has shown potential value in predicting curative effect, but the time and frequency of scanning, as well as the correlation between DWI parameters and tumor pathological response at different time nodes were not clear.

PDX models have recently emerged as ideal tools for the development of antitumor agents. In our study, we introduced this type of model to confirm the use of the ADC values in ESCC response prediction and assessment to nCRT. This type of xenograft model has been reported in previous studies. For example, Jiang et al. established 26 ESCC patient tumor-derived xenografts and found that the pathological characteristics of the P3 xenografts, which expressed CK5/6, p40 and p63, were consistent with those of the original patient samples (25). Additionally, Zhou et al. reported that 40 PDX models established from 188 esophageal carcinoma samples and all consecutive generations of P3 and P4 cells maintained the same histology as the primary tumors (26). We first successfully established PDX models from 4 patients with ESCC. Because the experimental techniques used to establish the PDX models were well established, only H&E staining and p63 expression were used for validation in our study. The validation assays demonstrated that the model histology remained similar between the passages and the original ESCC patient samples. Most importantly, H&E staining revealed interstitial tissues in addition to tumor cells; this finding benefited the development of our study and verified the reliability of the experimental results. In this study, we successfully established PDX models of ESCC from tumor tissues to confirm the use of the DWI in ESCC early pathological response prediction and assessment to nCRT, and could provide a theoretical basis for the clinical application of this technique.

We first investigated the effect of nCRT on ESCC PDX tumor growth. The tumor volume in the experimental group had a slight increase in the middle of the treatment, however, it shrank obviously and significantly smaller than that of the control group at the end of the observation period (*P*< 0.001). Meanwhile, the tumor transplanted in the control group showed a stable growth during the entire observation period and increased approximately double times as the initial volume at the end of treatment. The tumor transplanted in the experimental group had a slow growth in the middle of treatment, on one hand, due to the tumor tissue edema after chemoradiotherapy. On the other hand, its may could a secondary effect followed by chemoradiotherapy, which was named compensatory cell proliferation. That is, cells could undergo several mitotic divisions before dying. Subsequently, as the compensatory proliferation effect of tumor cells gradually disappeared, apoptotic signaling pathways were activated by radiotherapy, and tumor apoptosis occurred in tumor cells (27). During this period, cytolysis caused by chemoradiotherapy, tumor tissue necrosis, and removal of cellular debris would result in a slowing in tumor growth or even tumor shrinkage. Therefore, there was a significant difference in the tumor volume between the control group and the treatment group at the end of the observation period, furthermore, the differences in the △V_end-mid_ and △V_end-pre_ between these two groups also consistently reflected the growth delay of PDX models at the end of the treatment in our study.

Then, we investigated the effect of nCRT on ADC values of ESCC PDXs. The ADC values of the experimental group decreased obviously and significantly higher than that of the control group from the middle of the observation period (*P*< 0.001). In terms of microscopic functional metabolism, with the occurrence of tumor tissue injury caused by chemo-radiotherapy, the cell density decreased, and the dispersion ability of the water molecule would increase. As a result, the ADC value increased gradually from the middle of the treatment and reached the highest point at the end of the observation periods.

Furthermore, the result was well reflected in △ADC_mid-pre_, △ADC_end-mid_ and △ADC_end-pre_ values between the two groups in our study (all *P*< 0.001). In a word, the significant differences in the ADC volumes of transplanted tumors in the two groups were observed from the middle of treatment, however, the difference in the volumes was only observed at the end of the observation period. That is, the variation of ADC values preceded the changes in tumor morphological structures in our study. Our results are consistent with those of a previous study by Zhang et al. (28). They also proved that the variation of ADC values preceded the changes in tumor volumes. Thus, the observed variations in ADC values in our study appear to be in line with the early prediction and assessment to nCRT,

Based on the abovementioned results, we further evaluated and predicted the pathological remission response of ESCC after nCRT by ADC values and tumor volumes. We found that the change in ADC values before and middle treatment may be able to better predict pathological remission response and could identified tumors with or without pCR to nCRT. However, it did not show a significant negative correlation with the degree of residual tumor cells(*P*>0.05). Our findings were different from those of De Corelli et al. (29), they revealed that the change in ADC values before and after nCRT was significantly higher in the group with pathological remission response than in the group without pathological remission response using 32 patients with esophageal and esophagogastric junction cancer (P<0.05). What’s more, we found that the tumor volume after treatment also seemed to better predict the pathological remission response and could identified tumors with or without pCR to nCRT, and there was a negative correlation between tumor volume after treatment and pathological remission grade (P<0.05). Therefore, the observed variations in ADC values before and middle treatment in our study may able to identify tumors with or without pCR to nCRT at an early stage. These changes were prior to the changes of tumor volume after treatment.

To further verify pathological response to nCRT on tumor proliferation and apoptosis in ESCC, tissues from PDX mice were evaluated by TUNEL and immunohistochemical staining in this study. Apoptosis of tumor tissues was firstly evaluated *via* both TUNEL and apoptotic marker (Bax) immunoreactivity in all groups. In this study, we found that the apoptosis rate of the experiment groups increased the most in the middle stage of treatment, especially the groups with pCR, and the highest apoptosis rate occurred in the end of the treatment, and the result was well reflected in our study: the ADC values of the experimental group were significantly higher than the control group in the middle stage of treatment (*P* < 0.05). Furthermore, the two PDX models achieving pCR showed higher cancer cell apoptosis rate compared with the other two PDX models achieving pathological partial response in the both middle and end treatment. That is, TUNEL results provided strong evidence that the Xenografts achieved pCR, inducing the highest apoptosis rate, is likely to be benefit from treatment at the early stage.Additionally, the immunohistochemical staining results also showed that the levels of apoptotic marker(Bax) increased the most, and the levels proliferation marker(PCNA and Ki-67) decreased the most in the middle stages of treatment, especially in the pCR groups. At the same time, the two PDX models with pCR exhibited the highest levels of apoptotic marker (Bax), and lowest levels of proliferation marker (PCNA and Ki-67) in the both middle and end treatment. Collectively, these results suggested that nCRT began to exhibit significant antitumor activity by promoting the apoptosis and inhibiting the proliferation of ESCC cells from the middle stage of treatment, especially for the patients with pCR, and the most effective antitumor activity might occur in the end of treatment.

The advantage of this study was that several time points were settled in our experiment, as well as correlations were further verified with other potential biomarker expressing information on tumor proliferation and apoptosis. However, there were several limitations in the present study should be emphasized. First, due to the limitation of the surgical specimens and the low overall transplantation rate, the number of established PDX models was small. Another limitation was that ADC values measurement depended on the diffusion sensitivity coefficient (b- value), but there was still no unified standard for the selection of appropriate b-value for DWI in esophageal cancer at home and abroad (30, 31). In addition, the subjects of our study were all patients with ESCC. However, due to the infect that the incidence of esophageal adenocarcinoma (EA) has been increasing year by year in recent years, future studies should include as many cases of EA as possible, which could provide more rich and precise information for clinical practice.

In conclusion, we successfully established ESCC patient tumor-derived xenografts that maintained the pathological characteristics of the patients’ tumors. Our results in these models revealed that the ADC values could be used to determine the tumor’s response to nCRT, especially in the middle stages of treatment and before the tumor tissue morphology changes, and further, the ADC values were consistent with the potential biomarkers reflecting histopathological changes. Therefore, we suggest that radiation oncologists could refer to the ADC values in the middle stages of treatment when predicting the tumor pathological response to nCRT in patients with ESCC in the future.

## Data availability statement

The datasets presented in this study can be found in online repositories. The names of the repository/repositories and accession number(s) can be found in the article/supplementary material.

## Ethics statement

The studies involving human participants were reviewed and approved by the institutional research ethics board of the Shandong Tumor Hospital Ethics Committee (SDTHEC201911017). The patients/participants provided their written informed consent to participate in this study. The animal study was reviewed and approved by animal experimental guidelines of Shandong Cancer Hospital. Written informed consent was obtained from the individual(s) for the publication of any potentially identifiable images or data included in this article.

## Author contributions

YZ, JL, and JS: Design and conception of study materials, contribution to the data collection and assembly, and manuscript drafting. YZ and JS participated in analyzing and interpretating data, and in revising the content. All authors read and approved the final manuscript. All authors contributed to the article and approved the submitted version.
